# Psychiatric drugs dispensing trends in the affected population following Brumadinho dam failure

**DOI:** 10.3389/fpubh.2025.1507556

**Published:** 2025-05-16

**Authors:** Marcelo Farah Dell'Aringa, Gabriel Elias Correa-Oliveira, Francesco Della Corte, Luca Ragazzoni, Ives Hubloue, Virginia Murray, Thais Piazza, Claudia Garcia Serpa Osorio-de-Castro, Elaine Miranda, Francesco Barone-Adesi

**Affiliations:** ^1^CRIMEDIM - Center for Research and Training in Disaster Medicine, Humanitarian Aid and Global Health, Università del Piemonte Orientale, Novara, Italy; ^2^Neuroscience and Behaviour Department, Universidade de Sao Paulo, Ribeirao Preto, Brazil; ^3^ReGEDiM - Research Group on Emergency & Disaster Medicine, Vrije Universiteit Brussel, Brussels, Belgium; ^4^UK Health Security Agency, Global Disaster Risk Reduction, London, United Kingdom; ^5^Departamento de Gestão e Incorporação de Tecnologias em Saúde (DGITS), Secretaria de Ciência, Tecnologia e Inovação e do Complexo Econômico-Industrial da Saúde (SECTICS), Ministério da Saúde, Brasília, Brazil; ^6^Escola Nacional de Saúde Pública Sergio Arouca, Fundação Oswaldo Cruz, Rio de Janeiro, Brasil; ^7^Faculdade de Farmácia, Universidade Federal Fluminense, Niterói, Brasil

**Keywords:** mental health, public mental health (PMH), public health, pharmaceutical assistance, disaster medicine, public health data, mental health policy

## Abstract

**Introduction:**

In January 2019 D1 tailing Dam Failed in Brumadinho, Minas Gerais, Brazil. Two hundred and seventy people were immediately killed when 11.7 million cubic meters of mining byproducts were released, promoting major destruction and environmental damage traveling through the Paraopeba river basin. This study aims to investigate the impact of this disaster in the dispensing of psychiatric drugs.

**Methods:**

We evaluated monthly aggregated data from 12 months before to 12 months after the event from two data sources, one accounting for psychiatric drugs dispensed by private pharmacies and the other by public health services. We compared the median dispensing of benzodiazepines and antidepressants from the periods before and after the event using the Mann Whitney test and performed a visual analysis of line graphs from both datasets.

**Results:**

Data shows an increase of 294% in dispensing of benzodiazepines in the month following the event with a return almost to the baseline subsequently. When comparing the periods before and after the event the increase was not statistically significant, going from 16.03 to 20.60 daily defined daily doses (DDD) per 1,000 inhabitants (*p* = 0.07). In the private sector dispensing increased from 8.54 to 11.70 (*p* = 0.01), whereas in the public it went from 6.67 to 8.91 (*p* = 0.15). Data on the dispensing of antidepressants showed a statistically significant increase in the period following the event, going from 44.15 daily DDD per 1,000 inhabitants to 53.32 (*p* = 0.02). In the public sector it rose from 27.89 to 32.43 (*p* = 0.20), and in the private from 14.90 to 22.03 (*p* < 0.01).

**Discussion:**

We observed a peak in the dispensing of benzodiazepines in the month following the event drawn by the dispensing of diazepam in the public health sector. Dispensing of both benzodiazepines and antidepressants tended to be higher in the period following the event. Our findings should be taken carefully due to the nature of the data used for the study. This study can serve as a call for more evidence and local guidelines on acute psychiatric pharmacological care following disasters and for better integration of pharmaceutical assistance in disaster plans.

## Introduction

Located just 9 km from the municipal center of Brumadinho (state of Minas Gerais, southeastern Brazil), the Córrego do Feijão tailing dam (Dam B1) was originally developed as a tailing dam for an iron ore mine in 1976 and was already shutdown as a deposit of mining refuse on 25 January 2019, when it failed. It contained 11.7 million cubic meters of mining byproducts composed mainly of iron, aluminum, manganese and titanium. The tailing-rich mud reached the Paraopeba River basin, not before sweeping away important infrastructure installed downstream. Two hundred and seventy people, mostly dam workers, were killed and an area equivalent to 450 football fields along the river was covered by the iron mud (1, 2).

Disasters are intrinsically painful for individuals, families, and societies, with great associated burden. Research in disasters aims to obtain evidence and produce information that may lead to decrease not only of mortality, but also of morbidity in the affected populations while also reducing risk and promoting faster and consistent recovery (3). A growing body of evidence is being produced and contributing to more efficient and effective medical and operational response in such situations. However, research in the field still has major gaps, some of which are due to the inherent difficulties in performing research in the aftermath of a disaster, when the situation usually does not allow the application of robust research methods such as randomizing and using prospective data.

In addition, disaster sciences deal with a very broad field of research encompassing professionals from different backgrounds, different populations and events linked to very different hazards. The difficulty of performing research is also promoted by a lack of clear definitions and guidelines, the fact that information is spread over different and non-interoperable databanks, and that emerging scientific production is diluted in many areas such as emergency medicine, global health and environmental engineering (4). On the other hand, important initiatives have come up in recent years, aiming to establish research and operational standards in the disaster sciences. The World Health Organization (WHO) Emergency Medical Teams Initiative (EMT Initiative), the WHO Guidance on Research Methods for Health Emergency and Disaster Risk Management (WHO Research Methods for EDMR) and the Sendai Framework Hazard Terminology and Classification Review Task Team (Sendai Hazard Terminology Task Team) are examples of that. Moreover, The Sendai Framework for Disaster Risk Reduction (Sendai Framework), which was endorsed by the United Nations General Assembly in 2015 as one of the 2030 Agenda agreements, states that “*To enhance recovery schemes to provide psychosocial support and mental health services for all people in need”* is necessary to achieve one of its four priorities: “*Enhancing disaster preparedness for effective response and to “Build Back Better” in recovery, rehabilitation and reconstruction”*, whereas its predecessor, The Hyogo Framework for Action, did not mention mental health (5).

Disasters promote distress in the affected populations and, taking coping capacities to the limit, may trigger acute or long-lasting mental disorders, or exacerbate pre-existing psychiatric symptoms (6–8). Anxiety and depressive spectrum disorders and post-traumatic stress disorder seems to be highly prevalent in affected populations (9–12). Mitigating strategies for the short and long-term mental health morbidity are a priority for health-care workers, policy makers and other stakeholders involved in disaster recovery.

A cohort study conducted in Brumadinho's population from July to December 2021 evidenced a prevalence of depressive, PTSD and anxiety symptoms which was higher than in the Brazilian population (13). A report released in 2025 by Fiocruz Minas Gerais highlighted that the prevalence of diagnosed depression in teenagers and adults remained stable from 2021 and 2023, and still much higher than what is observed in the Brazilian population. Moreover, the prevalence of psychiatric symptoms observed in 2023 was higher than what was observed in 2021 (14).

These findings are in line with studies evaluating the prevalence of psychiatric symptoms following diverse other disasters. A systematic review evaluating the mental health consequences of the Fukushima disaster, including 79 studies, identified high rates of individuals tested positive for psychological symptoms (15). A systematic review and meta-analysis published in 2023, which studied mental health disorders following disasters linked to natural hazards, also found a positive relation between exposure to these events and the prevalence of mental health symptoms (16). The PRISMMA study, a survey conducted by the University of Minas Gerais with the affected population of the Mariana Dam Failure, a disaster that happened in the same region of Brazil in 2015, also found a high prevalence of psychiatric disorders (17).

It is important to notice that not everyone who is affected by a disaster will develop psychiatric disorders. It depends on the type of event, how the individual was affected by it, the presence of previous psychological conditions, local and cultural characteristic, socioeconomic status and secondary stressors (18).

Mental health disorders are among the most prevalent and burden-associated health conditions in the world (19), with important barriers to overcome for treatment delivery (20) and a major gap between the needs and the services provided (21), especially in low- and middle-income countries (22, 23).

In disaster situations there is need to expand mental health care (24) to increase local response capacity and adapt it to emerging unmet needs (3) and, understanding how mental health services utilization may be influenced by disasters is a paramount step to achieve it.

Psychiatric drugs dispensing is a corner stone of mental health services; therefore, monitoring dispensing data may provide useful information for developing preparedness.

In this study, we aim to investigate how the Brumadinho Dam failure may have influenced the dispensing of benzodiazepines and antidepressants, analyzing retrospective data from before and after the event.

## Methods

### Data sources

We extracted monthly aggregated non-identified data from two data sources: the National System for the Management of Controlled Products (SNGPC—Sistema Nacional de Gerenciamento de Produtos Controlados) (25), which accounts for pharmaceuticals dispensed by private pharmacies and the Brumadinho Municipal Pharmaceutical Services Coordination, which accounts for pharmaceuticals dispensed by public health services. We only had access to industrialized drugs from the SNGPC because data on compounding pharmacies were unavailable.

Data from SNGPC was extracted from an open access government platform and data from Brumadinho municipality was sent by the secretary of health with authorization of use for research purposes.

### Data processing

Authors GEC and MFD went through the list of pharmaceuticals with controlled distribution in Brazil issued by Anvisa (Brazilian Health Regulatory Agency) and identified which ones were of interest in the management of mental health conditions. The pharmaceuticals were then classified as benzodiazepines or antidepressants.

Data were extracted as number of packages (when tablets or capsules) or vials (when oral solution) with the description of the number of milliliters or dosage forms present in each one as well as the amount of drug (in milligrams) per milliliter or dosage form. The total amount of drug distributed per month in milligrams was obtained by multiplying number of packages/vials by the amount present in each. Then, we divided the total monthly amount of each drug by its Defined Daily Dose (DDD) as suggested at the Anatomical Therapeutic Chemical (ATC). The ATC/DDD system was proposed by the World Health Organization in 1981 as tool to guide drug utilization studies, which allows drug utilization comparisons (26).

We then divided the monthly DDD per estimated population per year as defined by the Brazilian Institute of Geography and Statistics (IBGE—Brazilian Institute of Geography and Statistics) standardizing per 1,000 inhabitants and dividing by 30 (27). Data will therefore be presented as DDD per 1,000 inhabitants.

### Data analysis

We analyzed the monthly aggregated data from two periods: 12 months before the event and 12 months after the event.

The normality of the data could not be assumed by visual inspection nor by the Shapiro Wilks test, therefore we compared the median rates of dispensing, using the DDD per 1,000 inhabitants, for antidepressants and benzodiazepines in the periods before and after the event and their Interquartile ranges (IQR). Line graphs were generated with the dailyDDD per 1,000 inhabitants of benzodiazepines and antidepressants also plotting the 95% confidence interval (95CI) as defined by Byar's method. When trends were visually identified line graphs detailing the source of data (public or private) and by specific drug were plotted, to evaluate which sector or drug was responsible for the overall trend. To compare the medians, we used the Wicoxon Rank-Sum Test and considered as statistically significant a *p* < 0.05.

We used Wilcoxon Rank-Sum Test as this is a conservative test that does not require data to be normally distributed. Regarding multiple comparisons (MC), as this was an exploratory analysis and the number of tests was reasonably small (i.e., 6 tests), we did not consider a correction for MC necessary. Indeed, different authors advised not using formal MC adjustment when the analysis targets only one focused research question represented by a few closely related statistical hypotheses or parameters, and all analyses and estimates are reported with equal emphasis and detail (28, 29).

Graphs specifically for the Private Sector and Public Sector for both classes of drugs and, a graph for Diazepam with the confidence intervals generated by the Byar's method can be seen in Appendix 1.

Data Analysis was performed using StataCorp. 2017. Stata Statistical Software: Release 15.

### Ethical considerations

We used anonymized secondary public health data therefore an ethical committee evaluation was deemed unnecessary by the secretariat of the Ethical Committee for Research of the University of Sao Paulo in Ribeirao Preto, according to Brazilian ethical guidelines (Resolution 510/16).

## Results

Data shows an overall non-statistically significant increase in the total dispensing of benzodiazepines in Brumadinho in the period after the event, going from 16.03 to 20.60 daily DDD per 1,000 inhabitants with a *p* = 0.07. Analyzing the private sector, the increase was statistically significant, going from 8.54 to 11.70 with a *p* = 0.01 whereas there was a non-statistically significant increase in the public sector, from 6.67 before the event to 8.91 after the event with *p* = 0.15. Data on the dispensing of antidepressants shows a statistically significant increase after the event, going from a median of 44.15 DDD per 1,000 inhabitants to 53.32 with *p* = 0.02 This change was influenced by both a non-statistically significant increase in the public sector, going from 27.89 to 32.43 with *p* = 0.20, and by a statistically significant increase in the private sector going from 14.90 to 22.03 with *p* < 0.01 (Table 1).

**Table 1:** Median defined daily doses dispensing of benzodiazepines and antidepressants drugs before and after the event by sector, with the *p*-values obtained with the Wilcoxon Rank-Sum Test.

**Drug class and dispensing sector**		**Pre**	**Post**	***p***
		**Median (IQR)**	**Median (IQR)**	
Benzodiazepines	Public	6.67 (3.51–9.91)	8.91 (6.54–10.18)	0.15
	Private	8.54 (7.53–10.64)	11.70 (10.16–12.53)	0.01
	Total	16.03 (12.64–21.20)	20.60 (17.72–21.61)	0.07
Antidepressants	Public	27.89 (21;00–21.14)	32.43 (25.04–37.64)	0.20
	Private	14.90 (13.66–19.22)	22.03 (19.98–22.65)	< 0.01
	Total	44.15 (36.22–49.85)	53.32 (48.80–56.81)	0.02

**Figure 1:**
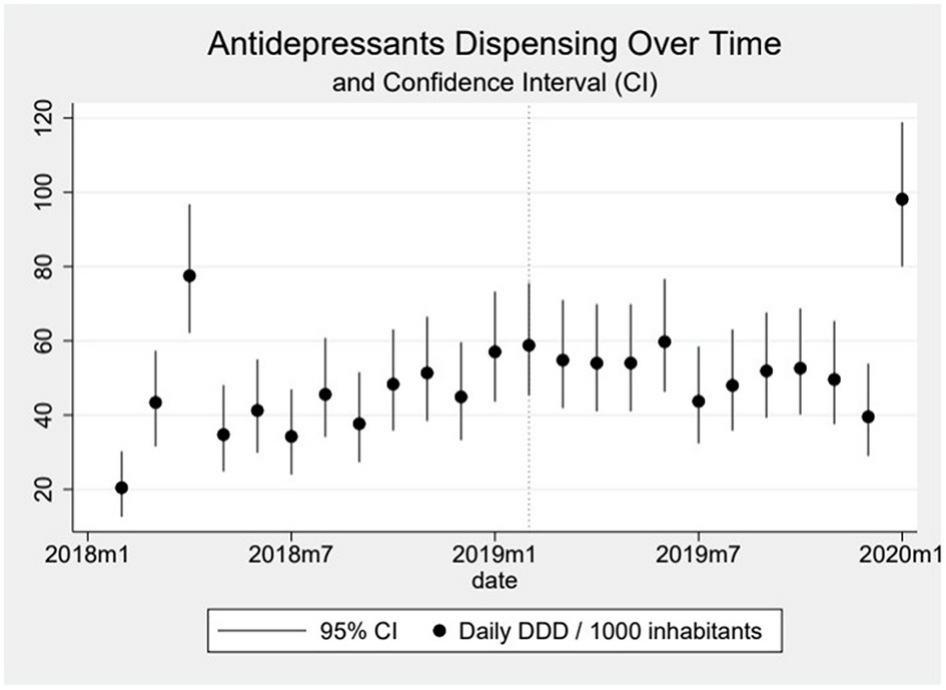
In the graph, the dots represent the daily DDD (Defined Daily Dose) of antidepressants dispensed over time per 1,000 inhabitants, and the vertical line over the dots the 95% Confidence-Interval calculated using the Byar's method. The dotted vertical line represents the moment the event happened.

**Figure 2:**
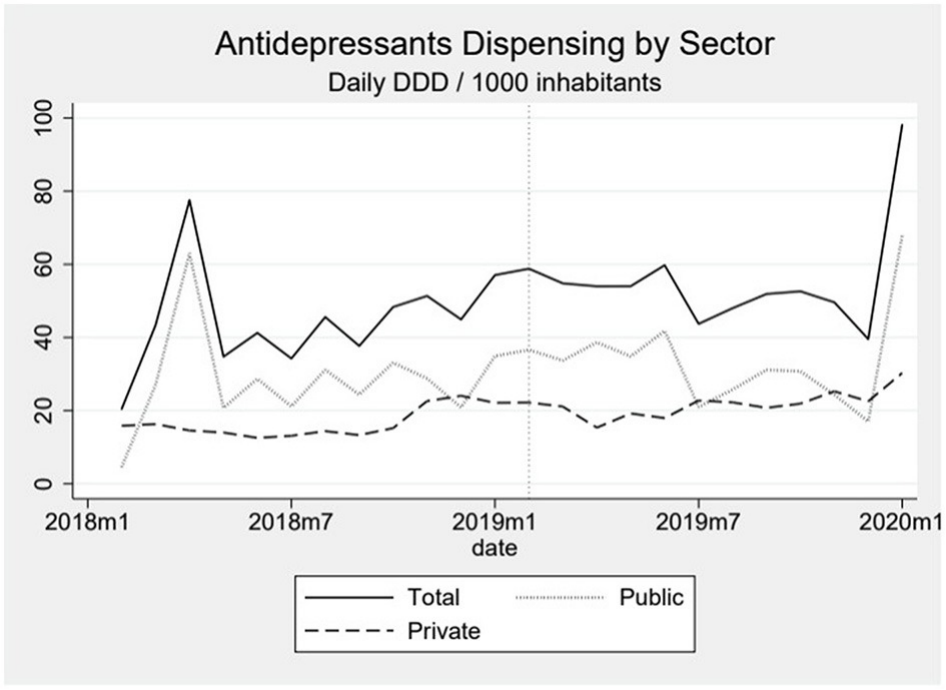
The graph shows the dispensing of antidepressants over time by sector, with the dotted line representing the public sector, the dashed line representing the private sector and the solid line the total. The dotted vertical line represents the moment the event happened.

Dispensing of antidepressants showed continuous growth throughout the analyzed series, without a clear relation to the event. In the beginning of the series, the total dispensing was around 35 daily DDD per 1,000 inhabitants whereas in the end it fluctuates around 50 (Figure 1). There are outliers in the first months of the series and in the last month and all of them seem to be caused by the rate of dispensing in the public sector. The first month of the series has a rate of almost zero followed by a peak of around 67, which led us to hypothesize that the inputs of a month may have been accumulated and entered in the following month (Figure 2).

**Figure 3:**
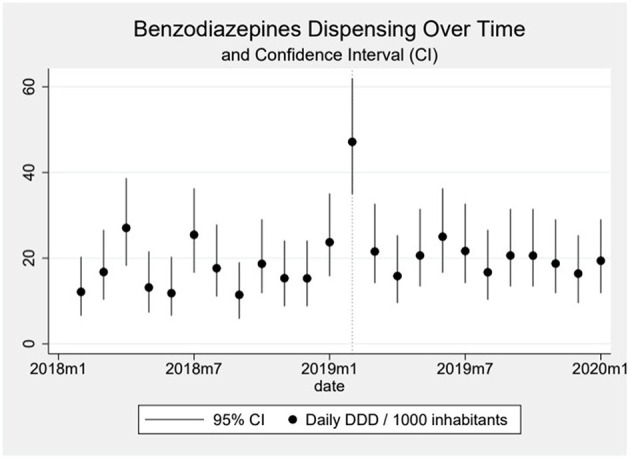
In the graph, the dots represent the daily DDD (Defined Daily Dose) of benzodiazepines dispensed over time per 1,000 inhabitants, and the vertical line over the dots the 95% Confidence-Interval calculated using the Byar's method. The dotted vertical line represents the moment the event happened.

**Figure 4:**
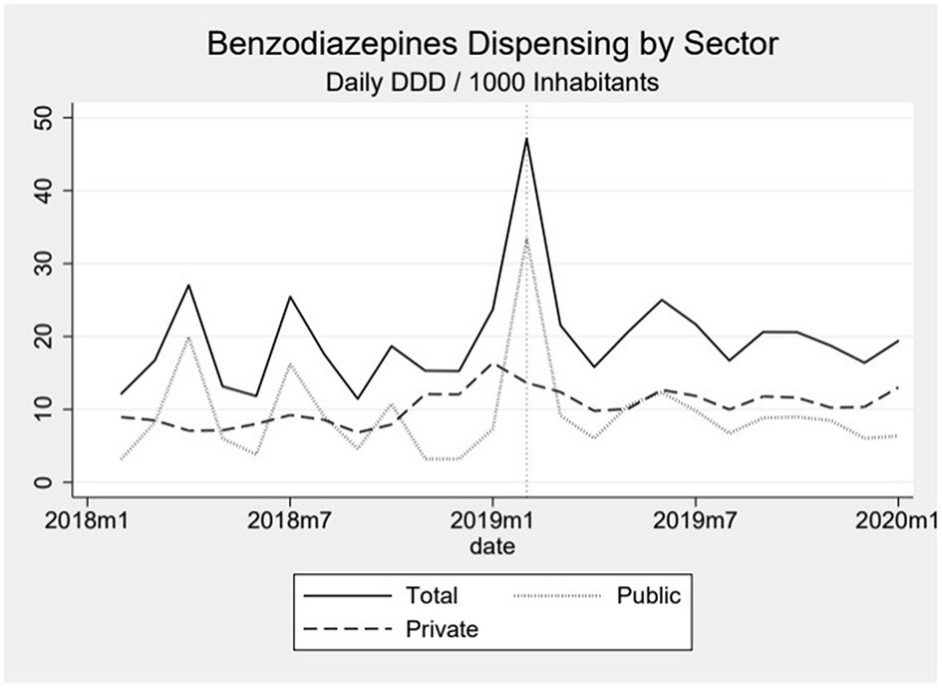
The graph shows the dispensing of benzodiazepines over time by sector, with the dotted line representing the public sector, the dashed line representing the private sector and the solid line the total. The dotted vertical line represents the moment the event happened.

**Figure 5:**
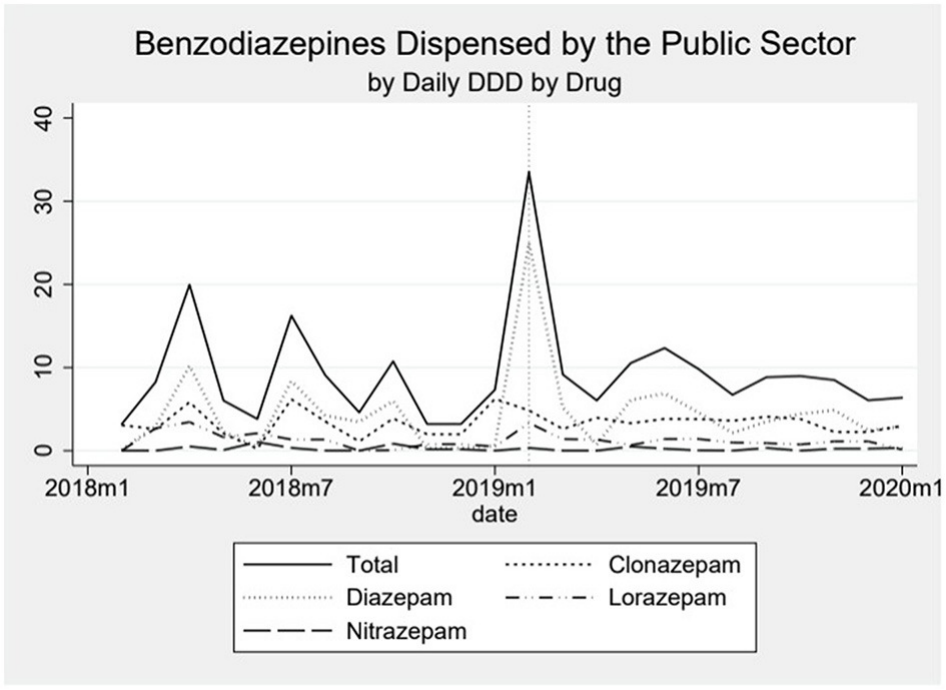
The graph shows the dispensing of benzodiazepines in the public sector over time by drug type. The dotted vertical line represents the moment the event happened.

Dispensing of benzodiazepines oscillated around 15 daily DDD per 1,000 inhabitants in the beginning of the series, reaching around 20 DDD per 1,000 inhabitants by the end (Figure 3). The oscillation seems to be influenced especially by the public sector, since in the private sector we observe a much smoother, almost linear increase in the first period and a plateau in the second period. We observe an important peak in the dispensing of benzodiazepines coinciding with the month of the event. This peak, an increase of 294% above the median pre-event, is mostly formed by a sharp increase in the public sector and dispensing goes back down, close to the baseline, shortly after (Figure 4). When analyzing data on public benzodiazepine dispensing by drug type, we observed that diazepam and clonazepam had the highest rate of benzodiazepine dispensing during the whole series and that the peak in benzodiazepine dispensing by the public sector is mainly formed by a peak in diazepam dispensing (Figure 5).

## Discussion

Disasters are traumatic experiences, causing acute distress in the affected population and the evolution to mental disorders in some individuals (18). Early systematic descriptions (30) already highlighted the presence of anxious, depressive, dissociative and hyper-arousal symptoms in affected individuals, just as corroborated by the modern diagnostic construct of Acute Stress Disorder (ASD) and Post-Traumatic Stress Disorder (PTSD) proposed by the Diagnostic and Statistical Manual of Mental Disorders (DSM) since it's 3rd edition. In addition, Major Depressive Disorder (MDD), Substance Use Disorders (SUD) and various other Anxiety disorders have higher prevalence in affected populations (31).

Our data shows a peak in the dispensing of benzodiazepines immediately after the event followed by a return to the baseline in the following few months. This finding is aligned with the findings of two studies performed in Holland. One is a prospective cohort study performed after the Volendam café fire (32) which happened in the New Year's Eve of 2000–2001. The authors analyzed the prescription of benzodiazepines for 4 years following the disaster, comparing data from the parents of the victims with a control group and evidenced that the parents of the victims had a higher utilization of benzodiazepines (OR 1.58) in the year following the event, which was not observed in the control group. When analyzing data from the 4 years following the event, there was no significant difference between the groups. The other study (33) is a longitudinal study, using retrospective data from 16 months before to 32 months after the event and analyzing data from the victims and from a comparison group. The authors of this study also evidenced a peak in the prescription of benzodiazepines but could not find convincing evidence that there was a prolonged prescription of benzodiazepines. A study evaluating psychiatric drugs dispensing after the Canterbury earthquake, in New Zealand, also evidenced a short-term increase in dispensing of anxiolytic and sedatives, which was not evidenced for antidepressants and antipsychotics (34). Their findings were similar when they studied the effects on older adults (35). Even though our findings are aligned with the ones from the abovementioned studies, care must be taken when comparing the findings since the methodology used is very different as are the time spam and time intervals.

In a study on populations which suffered from flooding in England the authors evidenced a higher prescription rate of antidepressants in the affected populations in the year following flooding (36). The difference between their finding and this study's may be due to the nature of the hazard studied but, it is also important to consider that the mental health impact of disasters does not translate directly into drugs prescribing or dispensing. We believe that one useful way of explaining it may be through Andersen's behavioral model for health services utilization which considers not only the needs for a service, in this case psychiatric drugs dispensing, but also predisposing and enabling factors (37).

Our study suggests that benzodiazepines were used to treat symptoms of acute stress in the immediate aftermath of Brumadinho dam failure. Data on the safety of the utilization of benzodiazepines for acute stress is scarce. Whereas, the prolonged use of benzodiazepines and their use as a treatment for PTSD is not indicated or strongly discouraged by current guidelines (38, 39) and by a meta-analysis (40); evidence on the use of benzodiazepines in the aftermath of potentially traumatic events (PTE) are much less robust. A systematic review and meta-analysis published in 2022 (41) studied the literature on the use of benzodiazepines in the aftermath of PTE and evidenced a trend toward a higher incidence of PTSD in those that used benzodiazepines. However, it only included 8 studies, two of which were trials published in 1996 and 2002, and the authors rated them as having a low methodological quality. In addition, differently from individual traumatic experiences, disasters are often followed by a period of increased risk of new disastrous events and injuries, such as the aftershocks in earthquakes, successive landslides following heavy rain or walking in rubbles after a hurricane. Benzodiazepines have a depressant effect on the central nervous system and may therefore reduce the response time to hazards and increase the risk of injuries in these situations.

Our data shows that the peak of benzodiazepines dispensing in the month following the event was mainly sustained by diazepam dispensed in the public sector. Even though diazepam is one of the most common benzodiazepines distributed by the public sector, we could not identify any specific local guidelines or strategy that explains such an abrupt increase in its dispensing. Further studies or a local evaluation of this finding could be useful for understanding this finding and subsequently proposing intervention strategies to guide prescribing. Notwithstanding, benzodiazepine use in Brazil shows a concerning trend, with lifetime exposure as high as 9.8% (42) and a noticeable increase in consumption specially by women, older adults and urban areas residents (43).

In parallel, there is evidence of recent growth in nationwide consumption of antidepressants (44). Two studies that evaluated private sector sales data between 2014 and 2020 showed antidepressant sales increasing from 13.7 to 33.6 daily DDD per 1,000 inhabitants in Brazil, driven by serotonin reuptake inhibitors and other newer medications (45, 46). This finding is likely influenced by a combination of factors such as mental health burden, socioeconomic conditions, demographic changes and, more recently, the impact of the COVID-19 pandemic (47, 48). The expansion of the mental health workforce in Brazil, as evidenced by the WHO Mental Health Atlas, may have increased awareness to mental health conditions and the rate of diagnosis, playing an important role (49).

In our study, antidepressant utilization both in public and private sectors maintained the same trends observed prior to the event, suggesting that prescription patterns of this drug class were not directly influenced by the event. It is noteworthy that many of these medicines are indicated for the treatment of PTSD, ASD and other anxiety reactions (38, 50–53).

Drug dispensing would ideally be integrated into disaster preparedness and response if disaster plans were to incorporate pharmaceutical services. Unfortunately, this does not occur today in Brazil. However, it may change, with the growing incidence of disasters and health emergencies, and studies that highlight this need (54–56). For feasible and responsible recommendations, considering only availability is not enough. Adequate municipal assessment of needs and services is paramount, and it cannot be achieved without adequate access to information. It is important to note that data from SNGP (25) was not available anymore from December 2021 but the SNGPC website states that the input of data on the platform will return to being obligatory in 2025.

Brumadinho tailing dam failure was the second of two major tailing dam failure in Minas Gerais, a State that holds 105 of the 905 tailing dams in Brazil (57). In 2015, Fundao Dam failure, in Mariana-MG, affected 13 municipalities producing major social economic impacts and environmental destruction. Vale S.A, the company that owned Brumadinho tailing dam also held an important role in the Fundao Dam. A previous study showed a trend of higher utilization of public mental health (PMH) services following the Fundao dam Failure and a study on psychiatric drug dispensing presented evidence of an immediate decrease in psychiatric drug dispensing followed by an upward trend in the long term. Comparisons between those studies and the present study need to be made carefully since time frames and methodologies were different and the previous did not use drug categorization. The authors from the previous study hypothesize that the immediate decrease in psychiatric drugs consumption may have been due to difficult access to these drugs, especially in the private sector, following the event (54).

This work can be useful highlighting an evidence gap in the acute care of psychiatric symptoms following disaster and bridging this gap is paramount for the development of guidelines on the management of these conditions. The peak in dispensing of benzodiazepines we evidenced in this study may have been due to overprescription or mismanagement, but without local previously agreed guidelines that's something we cannot affirm, moreover when evidence in the subject is still scarce. Aligned with what was found in previous studies, we did not evidence a long term sustained increase in the dispensing of these benzodiazepine, and some authors suggest this shows a lack of inadequacy in the prescription of this class of drugs, something we do not agree with. Even if the data we analyzed do not suggest the immediate peak in benzodiazepine prescription was translated in long term use and dependence, we cannot affirm it did not have impact in the immediate response to the event or to further development of PTSD and other long term psychiatric disorders.

## Limitations

Data from a previous study conducted in the affected population of Brumadinho evidenced that being female, living in the mining area, having 60 years old or more and having higher education levels were linked to a higher prevalence of psychiatric symptoms (13). In our study, due to the level of aggregation of data, we could not identify specific populations which may have influenced our findings.

Access and organization of the data sources were considerably different when comparing the public and private sectors. The access to data from privately dispensed controlled pharmaceuticals is publicly accessible through a federal platform where the data is inserted in a rather standardized way, whereas municipal data from Brumadinho may portray differences in notation. We believe this study may serve as a call to strengthen open access data, with more complex levels of aggregation. Even though the accesses to data from public institutions in Brazil is guaranteed by a law from 2011 (58) the interoperability of data has not been forthcoming. Such an initiative could follow or merge already existing platforms such as Datasus, where the user can already find aggregate data on health interventions, admissions and ambulatory care visits from the Brazilian Public Health System. We believe that implementing such a system or a single platform where data on the dispensing of controlled drugs, regardless of the sector where they are dispensed, would permit studies with more robust methodology and the use of comparison groups. In addition, it would make disparities in the access to psychiatric drugs between the public and private sector more promptly identifiable.

The nature of the data we analyzed did not allow for an evaluation of underlying trends in drug use.

We analyzed psychiatric drugs dispensing by the public and private services but after the event other sources of pharmaceutical dispensing may have been put into place. Vale, the company responsible for the dam, claims to have invested in healthcare professionals and pharmaceuticals after the event but we did not have access to this data. Therefore, data on dispensing after the event may be underestimated (59). As mentioned in the methods section we also did not analyze data dispensed in compounding pharmacies in the private sector. We do not deem this was an important source of dispensing benzodiazepines and antidepressants in Brumadinho and only 1 out of 11 pharmacies in Brumadinho (60) was working with compounded drugs at the moment of the event. However, we cannot exclude it contributed to the fact we did not find a relation between the dispensing of the drugs studied in the private sector and the event, underpowering our findings.

## Conclusion

We evidenced a non-sustained increase in the dispensing of benzodiazepines from the public health sector following Brumadinho dam failure. The higher dispensing rate of antidepressants evidenced in the period following the event is likely due to an underlying trend in higher consumption of this class of drugs in Brazil.

More studies are needed to understand the causes of the immediate peak in benzodiazepines prescription, to evaluate if the phenomenon is present in other disasters in Brazil and to analyse potential detrimental long-term consequences.

## Data Availability

The data analyzed in this study is subject to the following licenses/restrictions: Data on public sector drugs dispensing is available upon request. Data on private sector drugs dispensing is available upon request after authorization from the Brumadinho secretary of health. Requests to access these datasets should be directed to MD'A, mardellaringa@gmail.com.
