# Supplementary material for: Psychiatric drugs dispensing trends in the affected population following Brumadinho dam failure
Source: Front Public Health. 2025 May 16;13:1507556. doi: 10.3389/fpubh.2025.1507556 (PMC12124140; doi:10.3389/fpubh.2025.1507556)
Supplement: Supplementary file 1 [file Data_Sheet_1.docx]

**Appendix 1**


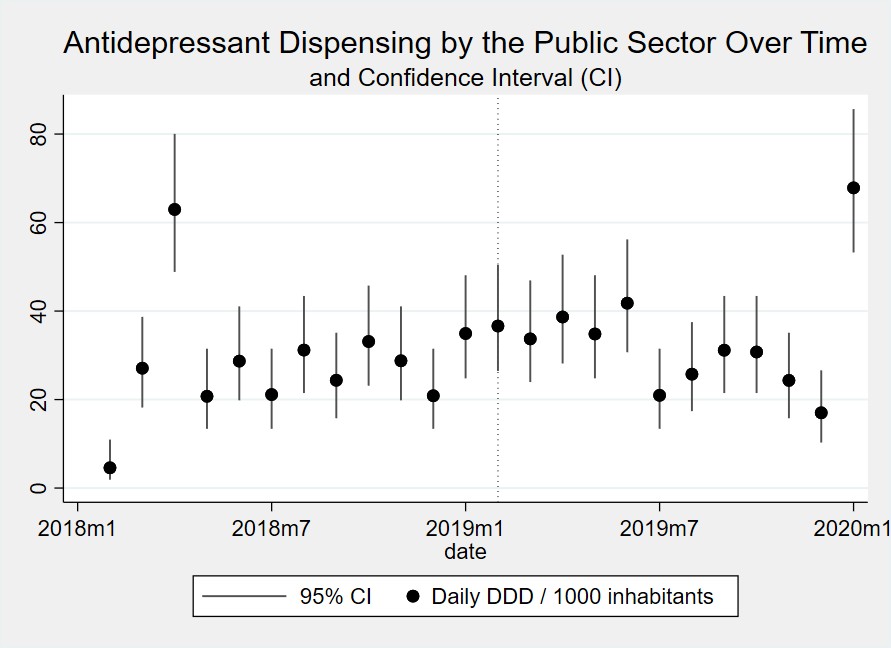


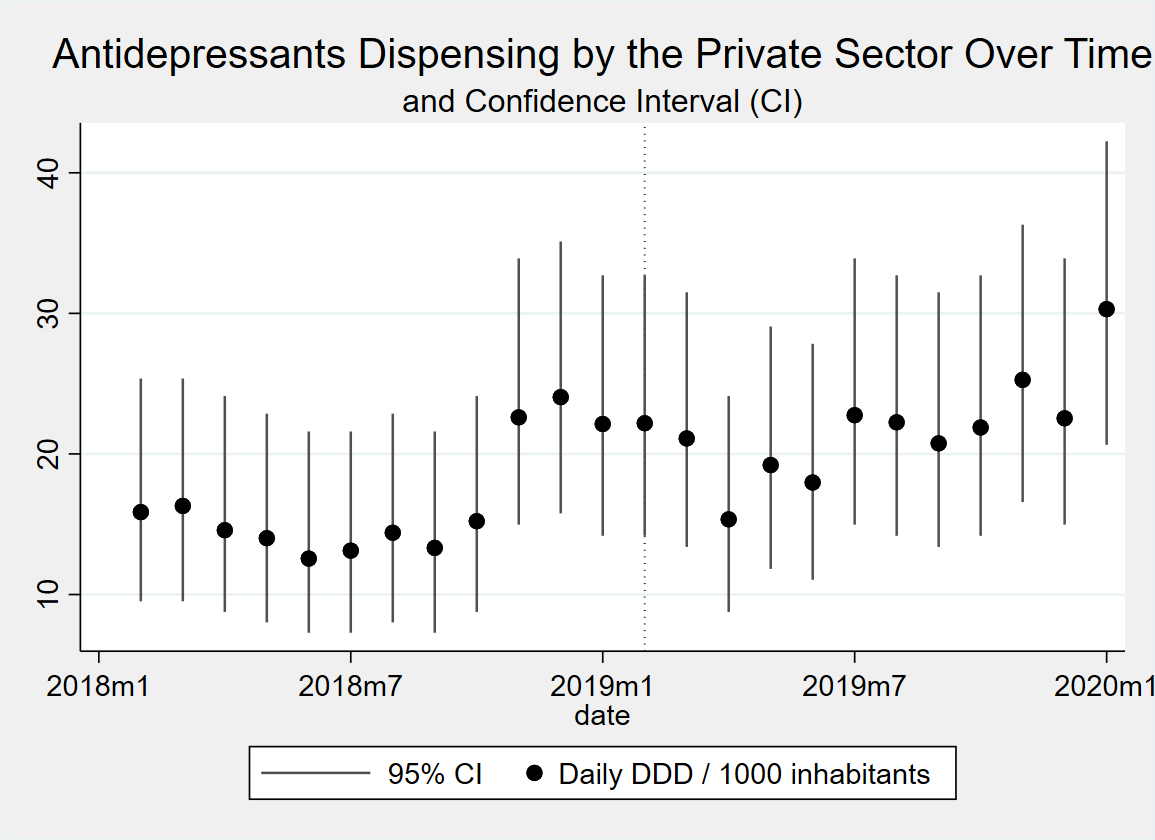


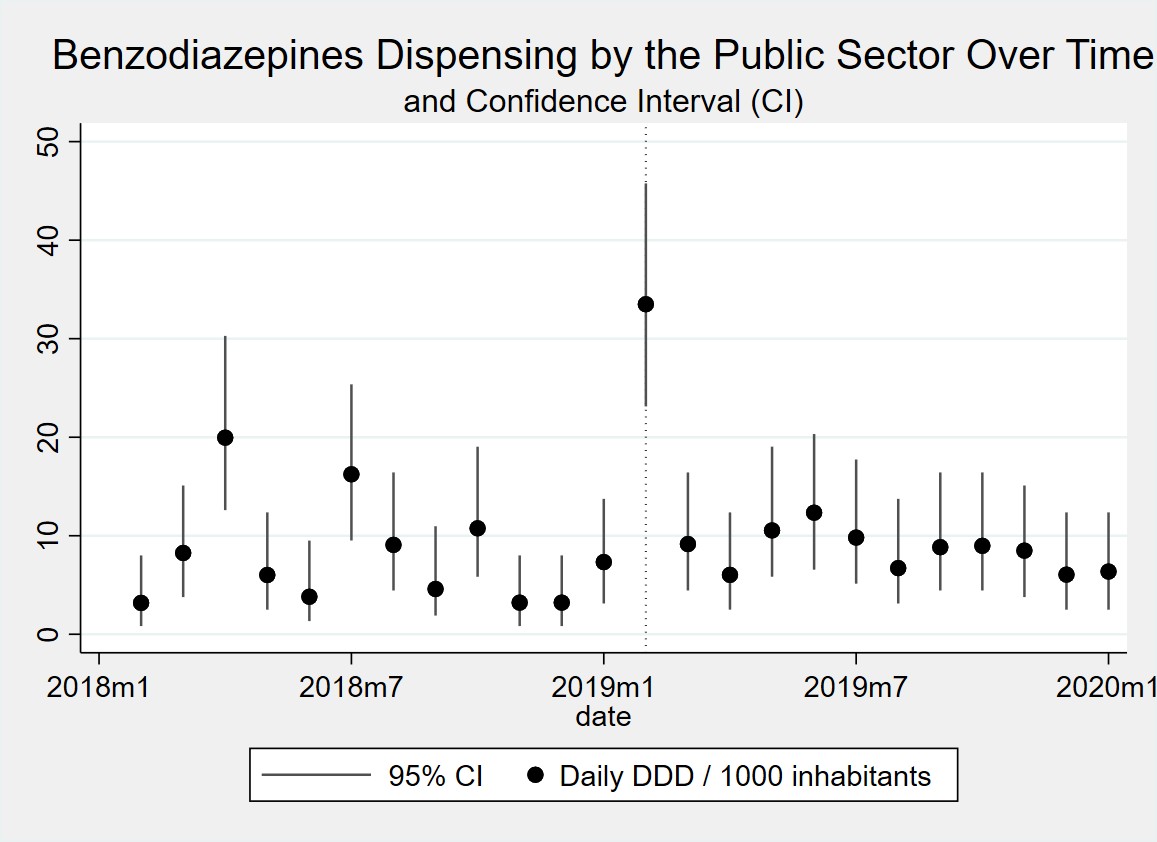


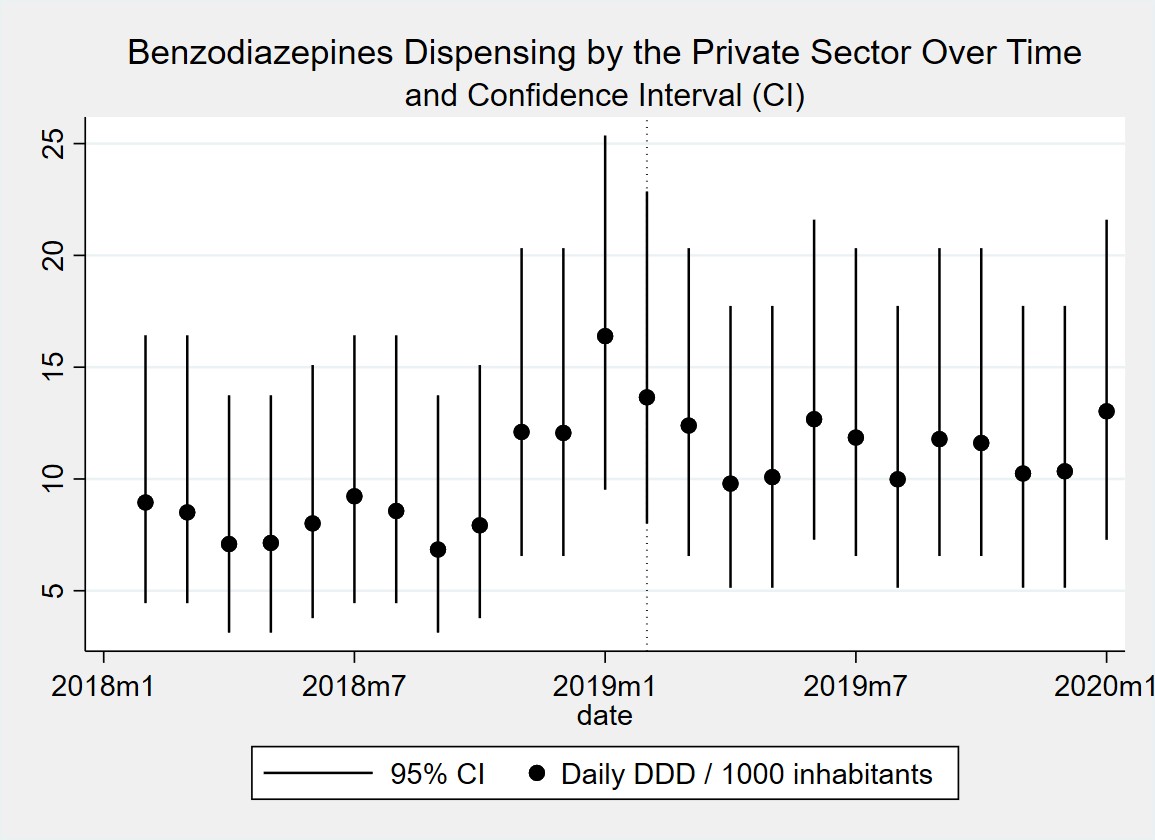


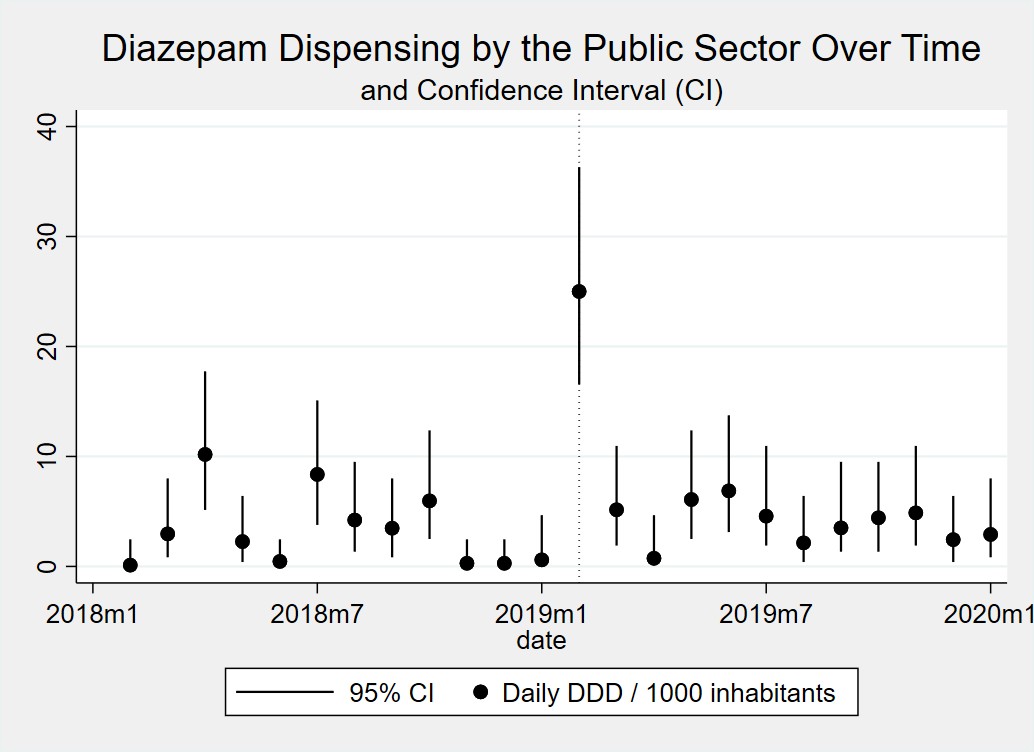


**Appendix 1 Artwork Caption**

Graph 6: In the graph, the dots represent the daily DDD (Defined Daily Dose) of antidepressants dispensed by the public sector over time per 1000 inhabitants, and the vertical line over the dots the 95% Confidence-Interval calculated using the Byar’s method.

The dotted vertical line represents the moment the event happened.

Graph 7: In the graph, the dots represent the daily DDD (Defined Daily Dose) of antidepressants dispensed by the private sector over time per 1000 inhabitants, and the vertical line over the dots the 95% Confidence-Interval calculated using the Byar’s method.

The dotted vertical line represents the moment the event happened.

Graph 8: In the graph, the dots represent the daily DDD (Defined Daily Dose) of benzodiazepines dispensed by the public sector over time per 1000 inhabitants, and the vertical line over the dots the 95% Confidence-Interval calculated using the Byar’s method.

The dotted vertical line represents the moment the event happened.

Graph 9: In the graph, the dots represent the daily DDD (Defined Daily Dose) of benzodiazepines dispensed by the private sector over time per 1000 inhabitants, and the vertical line over the dots the 95% Confidence-Interval calculated using the Byar’s method.

The dotted vertical line represents the moment the event happened.

Graph 10: In the graph, the dots represent the daily DDD (Defined Daily Dose) of Diazepam dispensed by the public sector over time per 1000 inhabitants, and the vertical line over the dots the 95% Confidence-Interval calculated using the Byar’s method.

The dotted vertical line represents the moment the event happened.
